# Research on Path Planning for Meniscus-Confined Electrodeposition on Non-Ideal Substrates with Global Inclination

**DOI:** 10.3390/ma19204312

**Published:** 2026-10-13

**Authors:** Jiangchuan Zhao, Pingsheng Xu, Hanchi Wan, Qiang Xing

**Affiliations:** School of Mechanical Engineering, Nantong University, Nantong 226019, China; 2510310008@stmail.ntu.edu.cn (J.Z.); 19305273633@163.com (P.X.); vans@stmail.ntu.edu.cn (H.W.)

**Keywords:** meniscus-confined electrodeposition, stable meniscus height, z-axis-weighted adaptive partitioning, traveling salesman problem path planning

## Abstract

**Highlights:**

- Hybrid ZAWP-TSP algorithm enables adaptive MCED path planning.
- Z-axis partitioning improves meniscus stability on uneven substrates.
- TSP optimization reduces idle travel and improves printing efficiency.
- SECCM-assisted plane fitting enables accurate 3D path mapping.
- Surface roughness below 0.05 μm demonstrates high-quality deposition.

**Abstract:**

To address the challenges associated with pattern deposition via meniscus-confined electrodeposition (MCED) on nominal-flat planar substrates with global tilt, including large variations in probe–substrate spacing, meniscus instability, and poor printing continuity caused by substrate inclination, a hybrid path planning algorithm, termed Z-axis-weighted adaptive partitioning and traveling salesman-based heuristic path optimization, is proposed. The algorithm integrates Z-axis-weighted adaptive partitioning with a traveling salesman problem (TSP) heuristic strategy, using the stable meniscus height as the partitioning criterion. Using the preset deposition pattern as input, this method extracts pattern boundaries, completes regional segmentation, fits the spatial plane of the substrate from four-point coordinates, and accurately maps two-dimensional (2D) image coordinates to three-dimensional space. The number of partitions is automatically determined under the constraint of the allowable height difference for a stable meniscus. After implementing Z-axis-weighted adaptive partitioning, a greedy nearest-neighbor TSP heuristic is applied to obtain a heuristically-optimized deposition sequence for each partition, reducing the path-switching time and improving the printing process stability. The experimental results demonstrate that the proposed method can achieve precision 2D pattern deposition of a 60 μm × 60 μm feature within an 70 μm × 70 μm planar area on conductive substrates with uncertain flatness. The printed trajectories are uniform with distinct line contours, and the surface roughness of deposited patterns is no more than 0.05 μm, indicating stable and reliable forming quality.

## 1. Introduction

Continuous advances in science and technology have deepened human understanding and exploration of the world. Consequently, research has progressively expanded from the macroscopic world to the microscopic realm [1,2]. Exploration of the microscopic world has evolved from early observations using microscopes to precision additive manufacturing at the micro- and nanoscale. Although modern three-dimensional (3D) printing technology is relatively mature at the macroscale, numerous technical challenges remain in micro- and nanoscale additive manufacturing [3,4,5]. Meniscus-confined electrodeposition (MCED) is inexpensive, widely applicable, and has broad applications for micro- and nanomanufacturing. Current research on microscale MCED has primarily focused on upward-grown copper micropillars and their arrays. Owing to the strong dependence of this technology on the meniscus morphology during printing, the direct fabrication of complex 3D structures remains challenging. Consequently, fabrication is achieved via layer-by-layer planar stacking [6].

To improve printing resolution and efficiency, extensive research has focused on optimizing MCED process parameters and system configurations. Early pioneering work by Iwata [7] demonstrated nanoscale metal deposition using microcapillary probes, achieving precise control over nanostructure dimensions. To scale up this capability, subsequent studies introduced porous embedded nozzles to maintain meniscus stability under sufficient backpressure [8]. Recent innovations have further enhanced the process by integrating dynamic cathode-scanning electrodeposition with electrowetting regulation to boost efficiency [9], as well as implementing dual-anode configurations in bipolar electrochemistry to mitigate bonding failures during dynamic transitions [10]. Prior MCED works mainly implement height compensation to stabilize the meniscus on uneven substrates [11], while 3D printing tool-path studies adopt traveling salesman problem (TSP) formulations to reduce nozzle idle travel without introducing meniscus physical constraints [12].

Few strategies simultaneously integrate meniscus stability constraints, region clustering and TSP sequencing.

Nevertheless, during practical planar deposition with glass microprobes, the uncertainty in deposition plane flatness can cause significant fluctuations in probe–substrate spacing, leading to meniscus instability and discontinuous or failed printing. In this work, we focus on the adaptive motion planning for nominally planar substrates with global inclination. Plane fitting is used to compensate for substrate tilt, which is validated on inclined planar gold-coated glass substrates. Therefore, motion planning that adapts to the substrate position is essential during probe movement to realize planar deposition of microstructures within a certain scale and provide algorithm references for future larger-size pattern fabrication.

In summary, adaptive path-planning algorithms must be integrated into existing experimental platforms to achieve continuous pattern printing via MCED. To address this challenge, this study proposes a hybrid deposition path-planning algorithm based on Z-axis adaptive weighted partitioning and the traveling salesman problem (ZAWP-TSP), combined with the positioning function of scanning electrochemical cell microscopy (SECCM). First, the deposition plane is scanned using SECCM, and plane fitting is adopted to obtain positional information and clarify the height distribution characteristics of the deposition area. Second, using Z-axis-weighted adaptive partitioning, the printing path points are divided into multiple subregions such that the internal height fluctuations within each subregion meet the meniscus stability requirements. The partitioning is performed using the stable meniscus height as the primary criterion and the allowable height difference for stable meniscus maintenance as the constraint, after which the centroid of each subregion is calculated. Finally, a greedy nearest-neighbor traveling salesman problem (TSP) heuristic is adopted to obtain a heuristically-optimized printing sequence of all subregions, thereby optimizing the overall printing path and improving printing efficiency. The proposed ZAWP-TSP innovatively couples meniscus stability constraints with Z-weighted region clustering and TSP sequencing. Unlike conventional plane compensation followed by raster scanning, which only maintains vertical height control and traverses all points sequentially [11], ZAWP-TSP partitions regions under the meniscus safe envelope and optimizes inter-region travel to reduce idle time [13].

## 2. Materials and Methods

### 2.1. Deposition Principle of MCED

**Figure 1:**
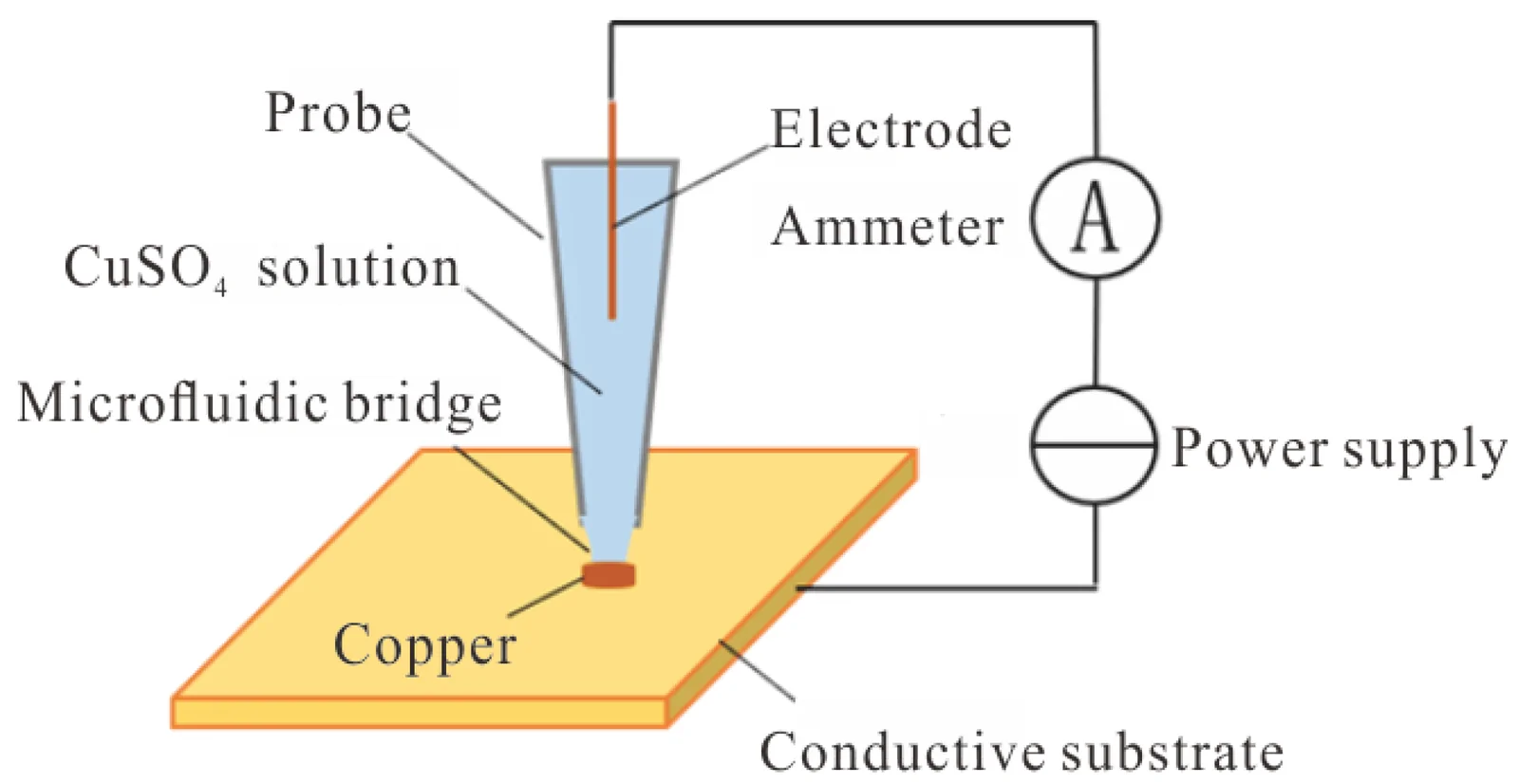
Schematic of MCED deposition.

MCED is an electrochemical deposition technique based on glass microprobes [14]. The deposition principle is illustrated in Figure 1.

First, glass capillaries are drawn into glass microprobes with micro-and nanoscale aperture diameters using a drawing instrument, and the resulting microprobes are filled with an electrolyte containing metal cations. Second, the glass microprobe is driven to approach the conductive substrate gradually via a high-precision piezoelectric driving platform, forming a stable microliquid bridge between the probe tip and the substrate. Once the microliquid bridge is established, the metal cations within the bridge undergo reduction reactions under an electric field, forming metal atoms that are deposited onto the conductive substrate. Through this process, target metal microstructures can be fabricated by the real-time regulation of parameters such as the liquid outflow rate, deposition current, 3D motion direction, and the displacement of the glass microprobe [15,16,17].

### 2.2. Image Preprocessing and Path Generation

**Figure 2:**
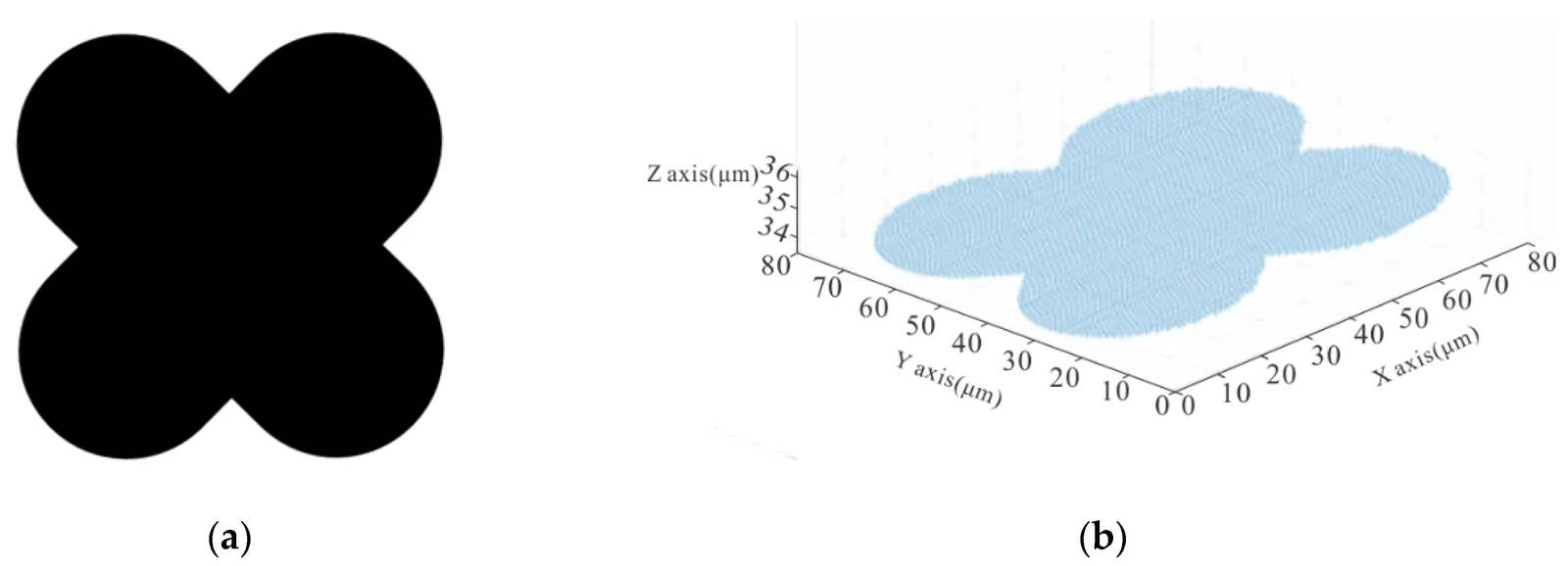
Schematic of the image preprocessing process: (**a**) Binary image and (**b**) Scanning path points.

Owing to the strong dependence of MCED printing on meniscal morphology, 3D microstructure fabrication must be realized through layer-by-layer stacking, with each planar layer printed using a region-filling algorithm. Typical region-filling algorithms include parallel scanning, zonal and fractal scanning, and contour-offset methods. Reasonably designed zonal parallel scanning filling strategies can shorten the deposition motion time and improve formation quality and deposition efficiency [18,19]. For pattern printing, binarization is first performed to obtain a binary image with the specified pixel dimensions, as shown in Figure 2a. The printing area is extracted in advance and used as the reference image for deposition. The binary image of the deposited pattern is defined as a discrete mapping function, as expressed in Equation (1).

*I_b_*:*Ω* ⊂ *N*^2^ → {0,1}. (1)

Here, *Ω* = {(*i*,*j*) ∣ 1 ≤ *i* ≤ *H*, 1 ≤ *j* ≤ *W*} denotes the image domain, where *H* and *W* represent the height and width of the input image, respectively. *I_b_*(*i*,*j*) = 1 represents the target deposition region, and *I_b_*(*i*,*j*) = 0 refers to the background region.

Owing to the uncertainties in meniscus morphology during deposition, the linewidth of the deposited structures tends to be unstable. Meanwhile, to better adapt the deposited patterns to the size of the deposition plane, the line width and spacing of the deposition points must be manually adjusted. Therefore, a simple line-scanning method was adopted, enabling the adjustment of the intervals between deposition points and the row spacing. The deposition path points generated by the line-scanning method first require scan-line sampling. The line spacing is defined as *d_l_*, and a set of scan lines is generated along the vertical direction of the image [20] as

*Y* = {*y* ∈ *N* ∣ *y* = 1 + *k* ⋅ *d_l_*, *k* = 0, 1, … ,|(*H* − 1)/*d_l_*|}. (2)

Feature extraction is then performed. The row vector *r* = [*I_b_*(*y*, 1), … , *I_b_*(*y*, *W*)] is extracted from each scan line *y* ∈ *Y*. The boundary difference vector *d* = *diff*([0, *r*, 0]) is computed. The starting point *S* and ending point *E* of the line segment are then determined according to Equation (3).

(3) $$\{\begin{array}{c} S=\left\{s/d(s)=1\right\}\\ E=\left\{e/d(e)=-1\right\} \end{array}$$

For each line segment interval [*s_i_*,*e_i_*], the deposition points are generated with a point spacing of *d_p_* as

(4) $$P_{y}=\underset{i}{\cup }\left\{\left(x,y\right)|x=s_{i}+k\cdot d_{p},\;k\in N,\;x\leq e_{i}\right\}.$$

Finally, the path-point set for the image is generated, as shown in Figure 2b.

### 2.3. Principles of Coordinate System Conversion

During the two-dimensional (2D) planar deposition of microstructures via MCED, the meniscus is small and unstable, imposing stringent requirements on the flatness of the deposition plane. In practical deposition operations, ensuring an absolutely horizontal deposition plane is challenging, which frequently leads to the tip fracture of glass microprobes, disappearance of the deposition meniscus, and consequent deposition interruption.

**Figure 3:**
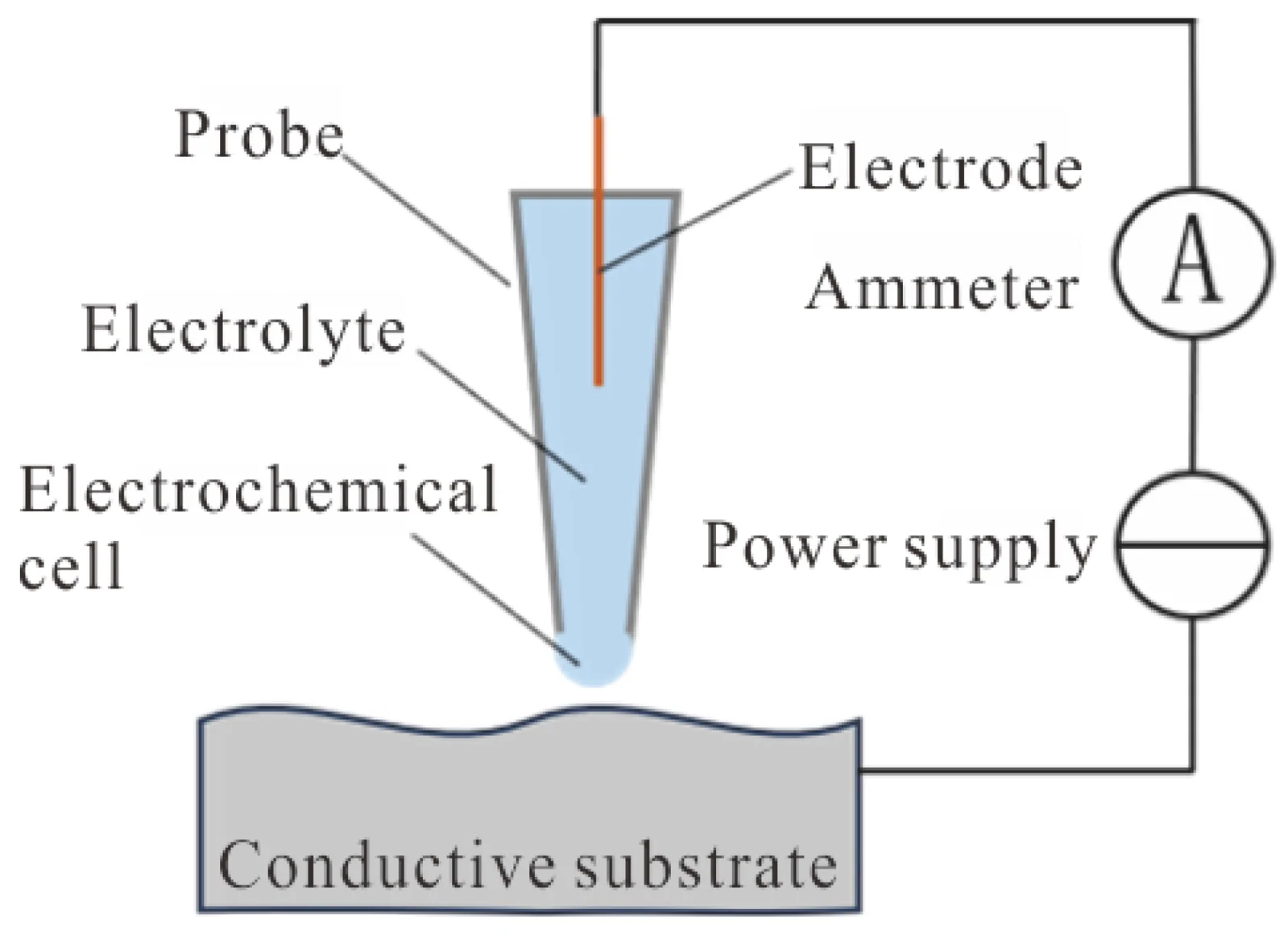
Schematic of the SECCM scanning principle.

As an advanced technique, scanning electrochemical cell microscopy (SECCM), proposed by Unwin et al. [21], enables high-precision topography measurement of sample surfaces by forming a tiny electrochemical cell between the glass microprobe tip and the sample under a bias voltage. This technique integrates electrochemical and microscopic scanning technologies. It can capture local variations on sample surfaces in real time and acquire high-resolution morphological information. Hence, it is widely applied in micro- and nanofabrication, surface analysis, electrochemical reaction research, etc. The working principle is illustrated in Figure 3.

Substrate flatness is often uncertain, and manual leveling using mechanical devices is time-consuming and insufficient to achieve high leveling accuracy. Therefore, the alternating-current scanning mode of SECCM is adopted to acquire the 3D coordinates of the substrate, followed by plane fitting to establish the plane equation of the entire substrate, enabling path conversion from the printed image space to the actual printing space.

To adapt to non-ideal flat substrates and maintain a constant probe–substrate Z-direction distance, the printing area adopts 3D spatial modeling. First, the piezoelectric ceramic drives the probe to the four corner positions of the deposition plane. An alternating voltage is then applied, and the glass microprobe is slowly lowered until it contacts the deposition plane, forming a tiny liquid droplet and generating a current feedback signal. Based on the signal, the height coordinates of the four points are obtained, thereby determining their 3D spatial coordinates on the deposition plane. Given the coordinates of the four non-coplanar corner points (*x_i_*, *y_i_*, *z_i_*), *i* = 1, 2, 3, 4, the least-squares method is used for plane fitting to establish the plane equation, as shown in Equation (5).

*z* = *ax* + *by* + *c* (5)

Next, an overdetermined system of linear equations is constructed [22], as shown in Equation (6).

(6) $$\left[\begin{array}{ccc} x_{1} & y_{1} & 1\\ x_{2} & y_{2} & 1\\ x_{3} & y_{3} & 1\\ x_{4} & y_{4} & 1 \end{array}\right]\left[\begin{array}{c} a\\ b\\ c \end{array}\right]=\left[\begin{array}{c} z_{1}\\ z_{2}\\ z_{3}\\ z_{4} \end{array}\right]$$

Let $A=\left[\begin{array}{ccc} x_{i} & y_{i} & 1 \end{array}\right]$ and *Z* = [*z_i_*]; the least-squares solution is solved as shown in Equation (7).

(7) $${\left[\begin{array}{ccc} a & b & c \end{array}\right]}^{T}={\left(A^{T}A\right)}^{-1}A^{T}Z$$

**Figure 4:**
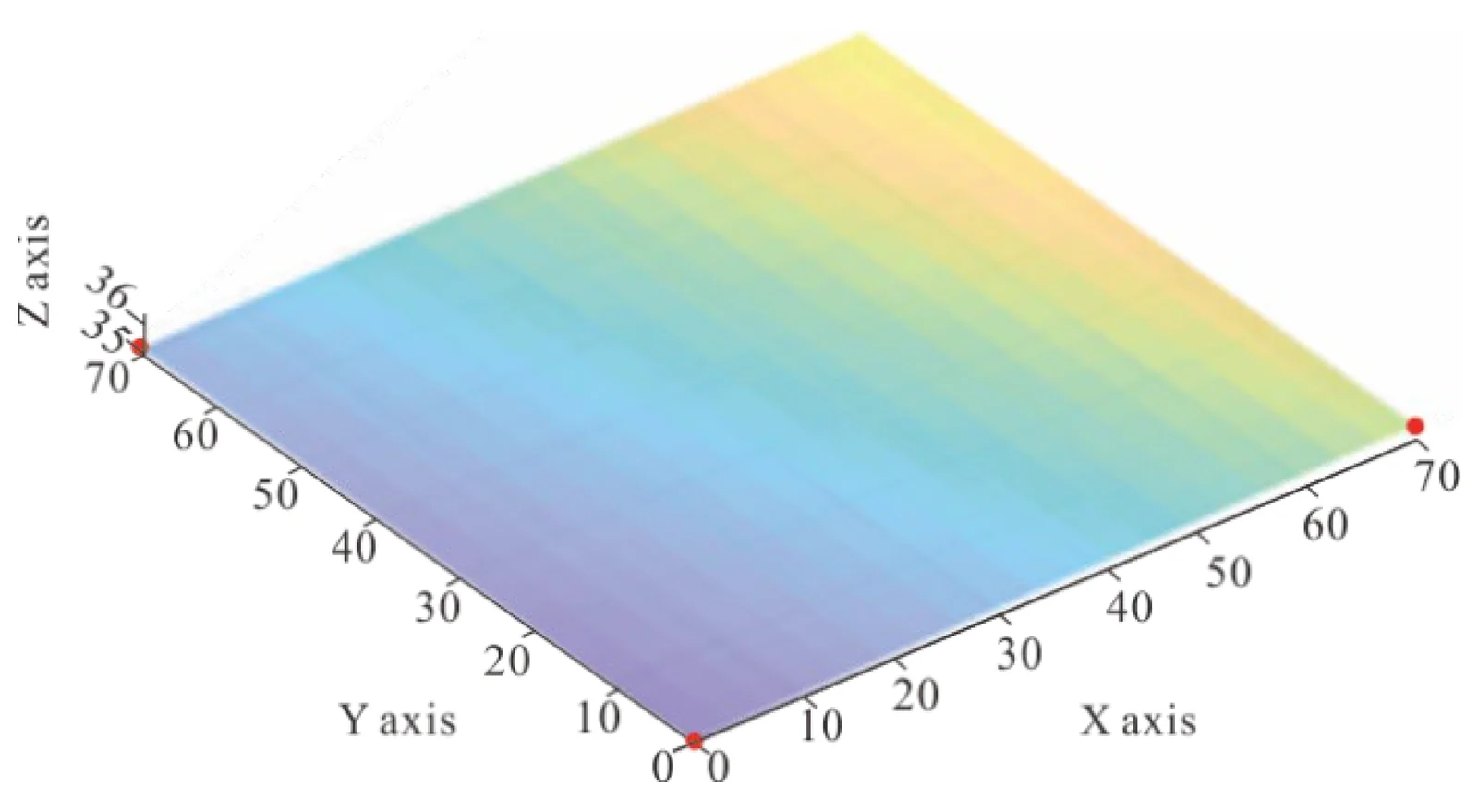
Schematic of the plane fitting process.

The obtained formula approximates the deposition surface model and is used for subsequent coordinate-height compensation and path projection. In the image-processing stage, all acquired deposition path points are expressed in the pixel coordinates of the printed image. To convert them into an actual coordinate system that matches the printing system, scale factors representing the actual physical length per pixel are defined. Subsequently, the 3D coordinates of the path points on the printing plane are calculated using the approximate plane equation model, enabling path conversion from the image space to the actual deposition space. The final deposition plane is shown in Figure 4.

### 2.4. Principle of Region Division

Although the fitted plane provides global Z-axis compensation for substrate tilt, it is merely an approximation of the real surface. Local substrate undulations, fitting residuals and measurement delays produce residual Z-axis deviations of approximately 0.5–1 μm. Full-field continuous scanning would accumulate such errors and may trigger meniscus rupture or probe collision. Besides, large-range XY displacement coupled with rapid real-time Z adjustment induces piezoelectric positioning jitter in MCED processes. Z-weighted partitioning aggregates points with comparable Z-features to constrain intra-region Z-adjustment ranges and mitigate transient disturbances to the meniscus. Without partitioning, frequent probe lifting-dropping between disconnected printing segments readily destroys the micro-liquid bridge. Partitioning enables continuous scanning within each sub-region and avoids redundant retraction. Further combined with centroid-based TSP optimization, it reduces inter-region travel distance and shortens printing time without sacrificing deposition quality.

The 3D coordinate data of the printing path points for the target binary pattern on the printing substrate can be derived based on the plane equation obtained by the plane-fitting algorithm described above and the scanning path points acquired via binary image preprocessing. Let the set of 3D path points be $P_{3D}={\left\{r_{i}\right\}}_{i-1}^{N}$, where ***r**_i_* = (***x**_i_*, ***y**_i_*, ***z**_i_*). If interpolation deposition is conducted directly after extracting the deposition path points, frequent start–stop, direction switching, and lifting and descending movements of the probe can easily cause fracture of the microliquid bridge between the glass microprobe and the deposition substrate. Therefore, to enhance printing stability, the deposition path points are divided into different regions based on the Z-axis coordinates. Region division is realized by increasing the weight of height features in clustering; the weighted feature vector is defined as follows:

*f_i_* = [*x_i_*, *y_i_*, *αz_i_*]*^T^*(*α* > 1) (8)

where *α* denotes the height weight coefficient.

The height-weighting factor α is optimized through numerical pre-experiments by scanning α in the range of 1 to 4. The optimal value α = 2.5 is selected under the constraint that the maximum height difference within every subregion does not exceed the meniscus-stable threshold Δ*H_allow_* = 1.08 μm, and with the objective of minimizing total printing time. A sensitivity analysis further shows that for the 70 μm × 70 μm planar area with Δ*H* = 2.02 μm, when α varies from 2.0 to 3.0, the partition count *K* remains 4, and the total printing time does not change significantly, indicating robust partitioning performance in this reasonable parameter range.

The weighted 3D path points are then divided into *K* consecutive square subregions constrained by the allowable height difference for a stable meniscus [23,24].

**Figure 5:**
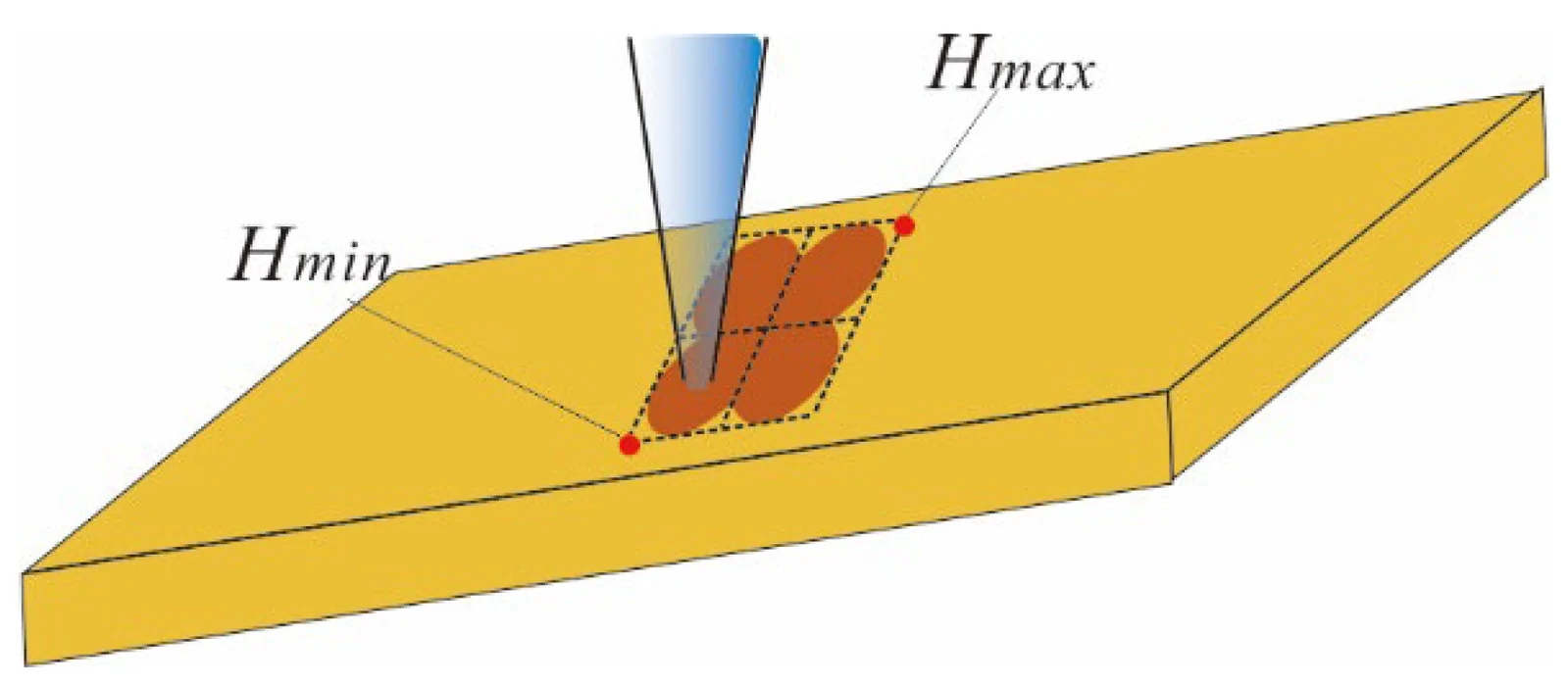
Schematic of height partition.

As shown in Figure 5, the number of divided regions *K* was automatically determined by calculating the ratio of the maximum height difference within the deposition area to the allowable stable height difference of the meniscus and rounding it. Because MCED is primarily used for planar 2D pattern deposition, its path points are distributed in a planar grid form. Adopting square zoning ensures a regular shape, uniform size, and unified boundary for each subregion, facilitating the subsequent regional centroid calculation and TSP path planning. Meanwhile, square zoning can evenly distribute the height fluctuation of the substrate such that the height variation within each subregion is well controlled. Under the constraint of an allowable stable height difference of the meniscus, uniform deposition stability across the entire area is guaranteed, avoiding local meniscus instability and deposition interruptions caused by irregular shapes and uneven sizes of the divided regions, thereby realizing continuous, stable deposition of planar patterns. It limits the height fluctuation within each subregion below the stable maintenance limit of the meniscus and prevents meniscus rupture and deposition failure caused by excessive fluctuations in the probe-substrate distance owing to substrate inclination. The formula is shown in Equation (9).

(9) $$K={\left\lceil \frac{\Delta H}{{\Delta H}_{\mathrm{allow}}}\right\rceil }^{2}$$

⌈⋅⌉denotes the ceiling function, which rounds the value upward to the nearest integer.

Here, *K* is the total number of square subregions on the 2D plane, ΔH represents the maximum height difference in the deposition area (Δ*H* = *Hmax*−*Hmin*), *Hmax* is the maximum height, *Hmin* is the minimum height within the deposition area, and Δ*H_allow_* stands for the allowable stable height difference of the meniscus. The optimal clustering partition is obtained by solving Equation (10).

(10) $$\underset{{\left\{C_{k}\right\}\;}_{k=1}^{K}}{\min}\sum_{k=1}^{K}\sum_{f_{i}\in C_{k}}{\left\|f_{i}-\mu_{k}\right\|}^{2}(\Delta H\leq \Delta H_{\mathrm{allow}},k=1,2,3\ldots ,K)$$

Here, *C_k_* represents the point set of the k-th region, Δ*H_k_* is the maximum height difference within the k-th subregion, and $\mu_{k}=\frac{1}{\left|C_{k}\right|}\sum_{f_{i}\in C_{k}}f_{i}$ denotes the centroid of each region.

The zoning method, based on weighted Z-axis coordinates, divides the printing points of the deposited patterns into adaptive regions. This prevents meniscus fracture and probe collisions with the deposition plane caused by excessive Z-coordinate differences and significantly improves the stability and continuity of deposition.

#### 2.4.1. Calculation Principle of the Allowable Stable Height Difference of the Meniscus

The stable morphology and allowable height difference of the meniscus are jointly determined using the Laplace capillary equilibrium equation and the three-phase interfacial energy.

**Figure 6:**
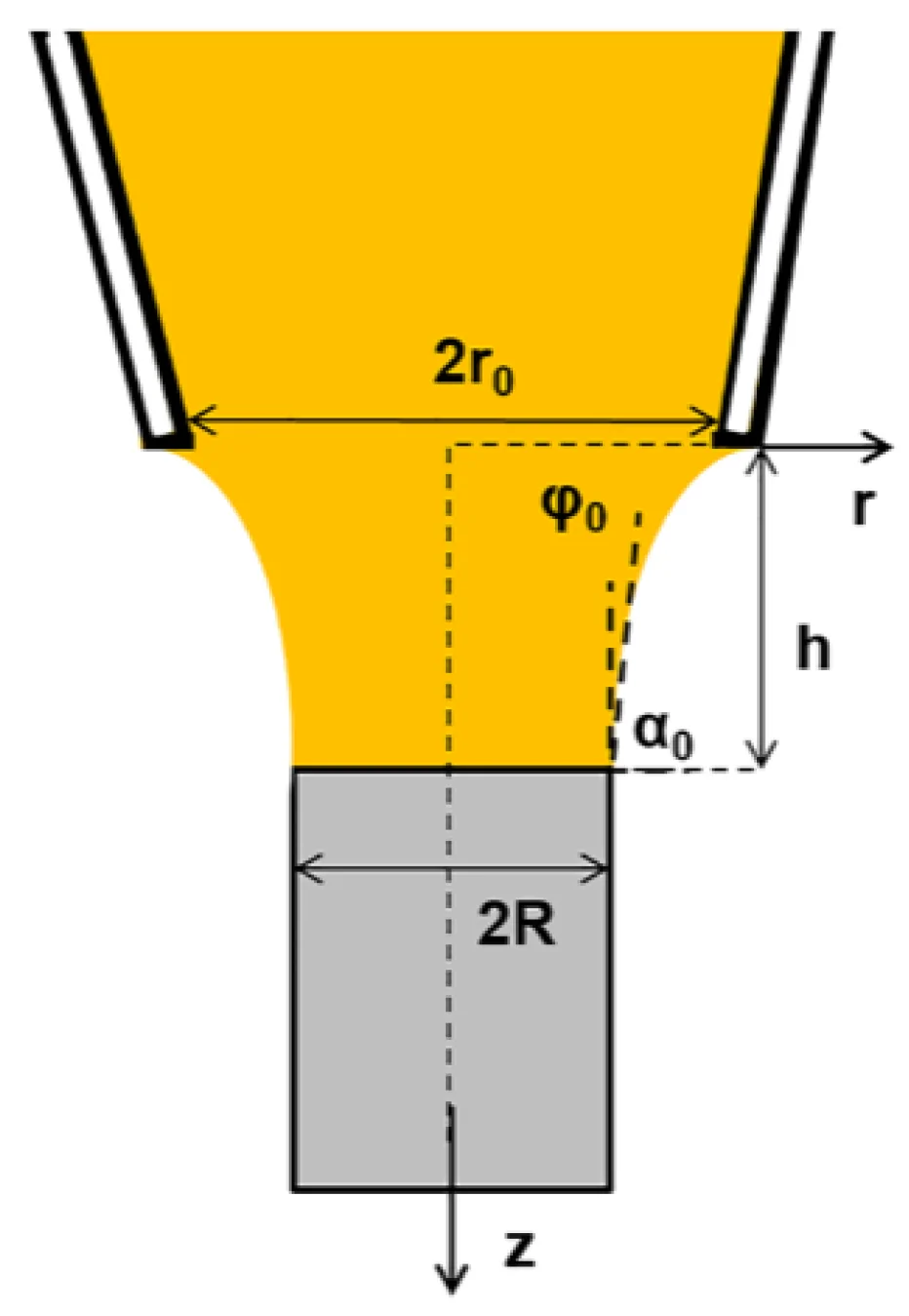
Schematic of the meniscus geometric model.

As shown in Figure 6, during the MCED deposition process, the meniscus height is a key geometric parameter for maintaining continuous deposition and a uniform structure, and its stable range must satisfy the following thermodynamic equilibrium and capillary mechanics constraints.

(11) $$z(r)=R\;{\sin\alpha }_{0}\left(\mathrm{arccosh}\frac{r_{0}}{R\;{\sin\alpha }_{0}}-\mathrm{arccosh}\frac{r}{R\;{\sin\alpha }_{0}}\right)$$

Based on the interfacial energy characteristics of the copper-water-air three-phase system, as described in Equation (11), the equilibrium growth angle was calculated to be approximately 12°. By solving the differential equation for the meniscus profile, the stable growth condition was obtained, indicating that the maximum allowable stable height of the meniscus should not exceed 27% of the nozzle diameter [25]. This 27% ratio is a condition-specific empirical threshold rather than a universal constant; its validity for other probe diameters, electrolyte compositions, and environmental conditions requires further systematic validation. Once this limit is exceeded, the liquid bridge becomes prone to instability and fracture, resulting in uneven deposition morphology and growth interruption. Within this range, the meniscus maintains a stable curved surface morphology. Combined with an ambient humidity of 60–80%, stable matching between ion convection and the deposition rate is achieved, ensuring the uniform growth of micro- and nanostructures.

#### 2.4.2. Principle of Trajectory Optimization

**Figure 7:**
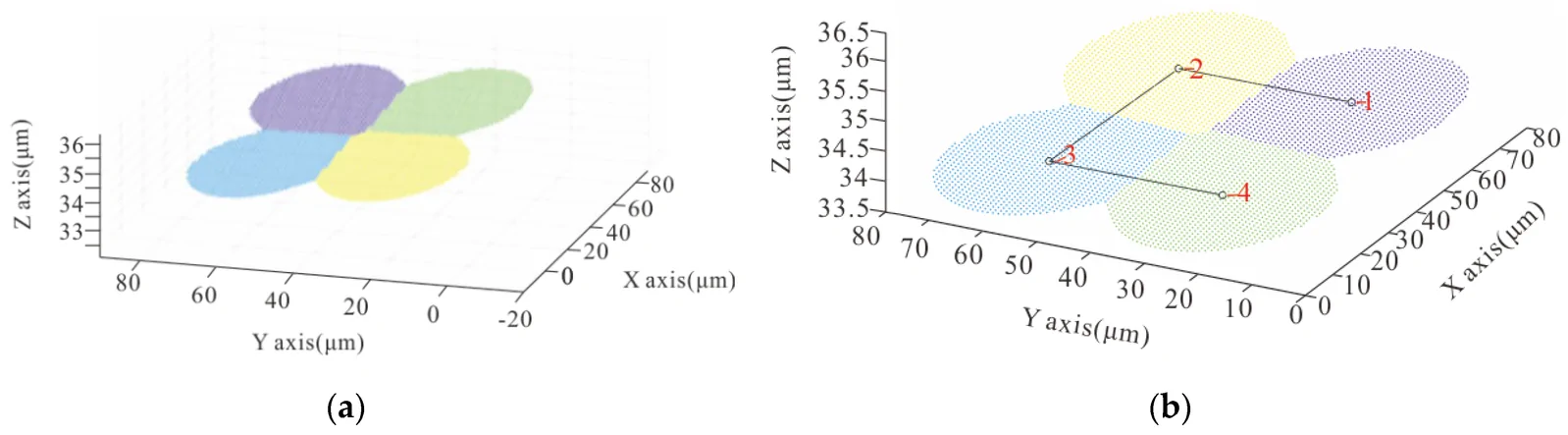
Schematic of height zoning. (**a**) Image zoning; (**b**) Printing sequence.

To ensure the complete deposition of elemental copper during printing, the moving speed of the glass microprobe must be strictly controlled, which leads to a long time consumption for MCED deposition. Direct deposition following the original path sequence easily causes redundant probe movement routes, and trajectory optimization is performed on the printing sequence for the divided regions to improve the deposition efficiency of the glass microprobes. Specifically, regional centroids are regarded as TSP nodes, and the greedy nearest-neighbor algorithm is adopted to generate initial solutions and to heuristically optimize the movement order of the glass microprobe [26] to minimize idle travel distance. Figure 7b shows the optimized printing sequence after zoning. Here, Δ*H* = *Hmax* − *Hmin* = 36.55 μm − 34.53 μm = 2.02 μm.

Suppose the centroid set of *K* regions is ${\left\{\mu_{k}\right\}}_{k=1}^{K}$. The nearest-neighbor search method is adopted. Starting from the initial point, the nearest unvisited point is sequentially selected until all points are traversed, yielding the heuristically optimized traversal order π^*^ that minimizes the total moving distance, as shown in Equation (12).

(12) $$\underset{\pi }{\min}\sum_{i=1}^{K-1}\left\|\mu_{\pi \left(i+1\right)}-\mu_{\pi \left(i\right)}\right\|$$

The traversal order of the regional centroids is heuristically optimized via TSP, which significantly shortens the travel distance between regions for the glass microprobes. The optimized deposition path forms a unidirectional continuous scanning trajectory, avoiding abrupt acceleration changes caused by frequent starts and stops.

The planar pattern deposition method based on glass microprobes effectively solves the probe–deposition plane matching problem through feature-driven adaptive zoning. Meanwhile, greedy nearest-neighbor TSP heuristic optimization of the guided trajectory planning achieves an evident improvement in efficiency and reduction in deposition time. It provides an algorithmic foundation for potential layer-by-layer 3D-microstructure fabrication via MCED, while its practical 3D-printing performance remains to be further validated through multi-layer experiments [27].

For the test mode adopted in this paper (dividing K = 4 subregions within a 70 μm × 70 μm planar area), we compare the solution obtained by the greedy nearest neighbor algorithm with the globally exact optimal solution of the Traveling Salesman Problem (TSP) derived by exhausting all 12 non-redundant traversal sequences. The total inter-region movement distances of the heuristic algorithm solution and the exact optimal solution are both 105.00 μm. This indicates that for the uniformly distributed subregions in this paper, the nearest neighbor heuristic algorithm can yield a global optimal result with no extra movement distance. It provides a low-complexity path planning algorithm for real-time printing in microscale microchannel electrochemical deposition (MCED).

## 3. Experimental Apparatus and Methods

### 3.1. Experimental Platform

The microadditive manufacturing platform adopted in this research consists of multiple modules, including microprobes fixed on the probe carrier, conductive substrates, and their carriers, XYZ 3D manual fine-tuning platforms, Z-direction micromotor coarse adjustment modules, XYZ 3D piezoelectric ceramic precision adjustment modules, piezoelectric controllers, signal collectors, ion current amplifiers, main controllers, and control hosts.

**Figure 8:**
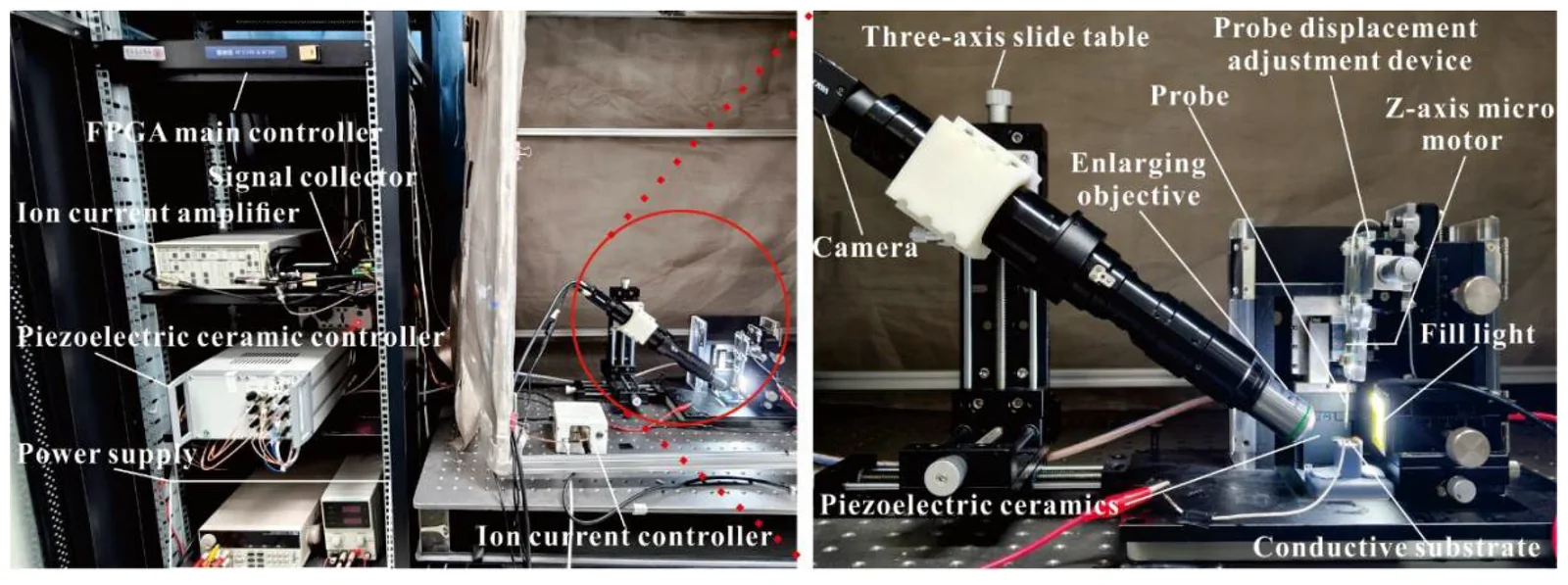
Experimental platform.

The Z-direction coarse adjustment device is composed of a Z-direction micromotor and its controller and is primarily used for rapid vertical positioning of substrates or probes. The XYZ precision adjustment module consists of piezoelectric ceramic actuators and their corresponding controllers to realize high-resolution, precise 3D positioning. A photograph of the adaptive planar microfabrication platform is shown in Figure 8.

### 3.2. Experimental Conditions

Electrolyte: A copper sulfate solution at 0.1 mol/L was prepared by dissolving copper sulfate pentahydrate (CuSO4·5H_2_O) in deionized water. At room temperature, solutions at this concentration rarely produce crystal precipitation, ensuring the smooth implementation of the deposition experiments.

**Figure 9:**
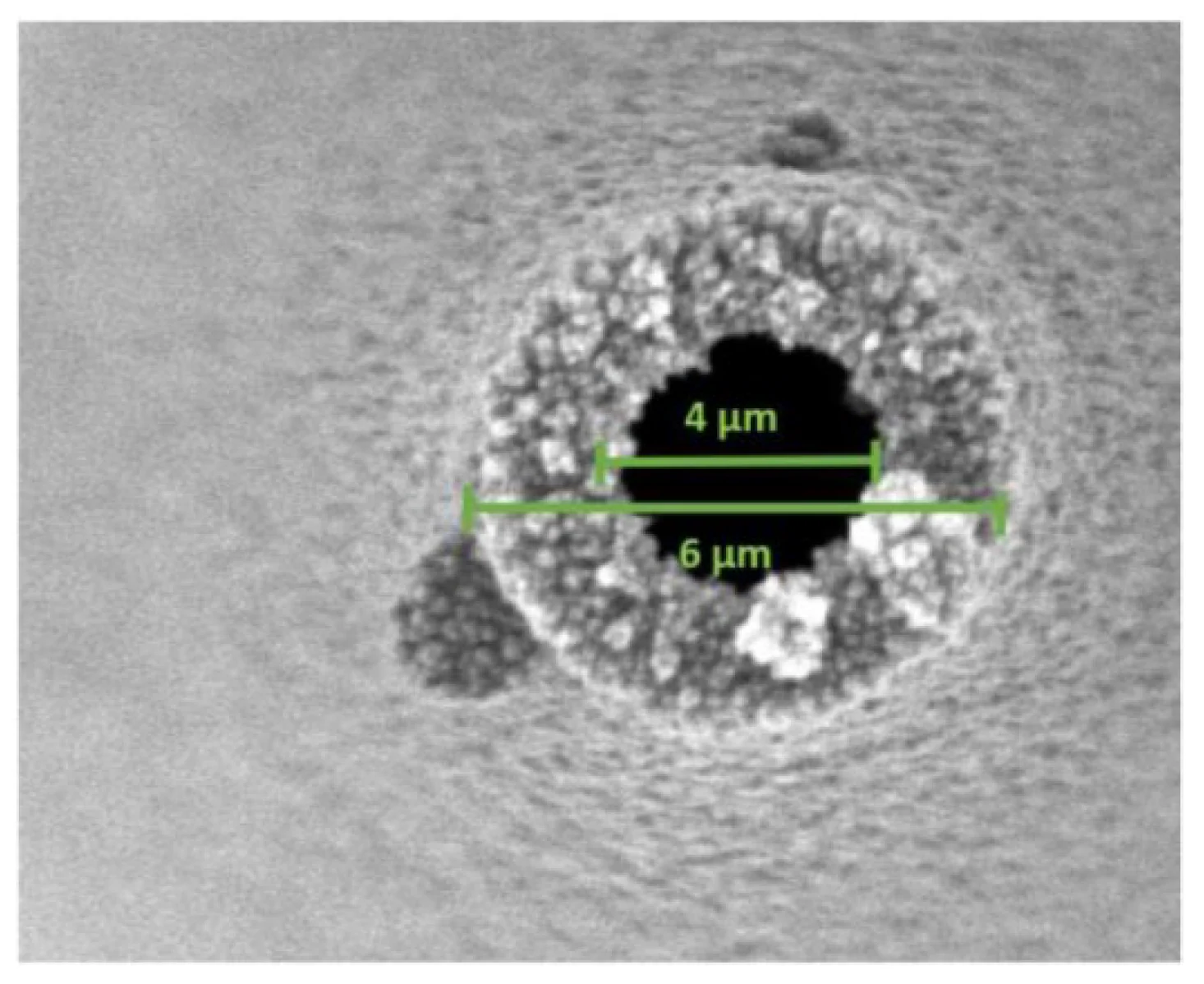
SEM image of the glass probe tip.

Glass microprobes: The glass microprobes used in the deposition experiment were purchased from Sutter Instruments, Novato, CA, USA. They were fabricated from BF100-58-10 borosilicate glass tubing using a microelectrode puller. During pulling, the heating temperature was set to 560 °C, and the pulling velocity was set to 20. As shown in Figure 9, the inner diameter of the probe tip was approximately 4 μm.

Deposition substrate: A 200 nm-thick gold film was sputtered onto a conductive glass with dimensions of 10 mm × 10 mm × 2 mm by magnetron sputtering. Conductive copper foil tape was used to connect the deposition electrode to the substrate, forming a complete conductive loop.

Microscope system: An industrial camera (MV-CA060-10GM Hikrobot, Hangzhou, China) equipped with a 20× metallographic objective lens and an LED backlight supplementary lighting system was used to observe the entire deposition process.

Characterization equipment: An inverted metallographic microscope (XJL-20, Liss Optical Instrument Co., Ltd., Guangzhou, China) was used to observe the surface deposition morphology, and a high-resolution confocal microscope was used to measure the surface roughness.

In the experiment, 0.1 mol/L copper sulfate solution was injected into glass micropipettes with a tip diameter of 4 μm, into which inert electrodes were inserted. The assembly was connected in series with an SRS-SR570 low-noise ion current amplifier and then linked to the positive terminal of the voltage regulator, which served as the anode for electrochemical deposition. A gold-coated conductive glass was connected to the negative terminal and acted as the cathode. Before each experiment, the surface of the inert electrode was polished to maintain smoothness and cleanliness and to prevent bubble retention during electrode insertion. In addition, a secondary liquid injection was performed after the first electrolyte filling to eliminate air bubbles within the microprobe, ensuring reliable experimental conditions.

### 3.3. Plane Fitting Verification Experiment

During planar microstructure printing using MCED, the uncertainty in the flatness of the deposition substrate often causes discontinuous deposition, leading to failure. Combined with the positioning characteristics of SECCM and the plane-fitting algorithm, the spatial plane equation of the deposition substrate can be calculated, laying the foundation for mapping subsequent deposition points.

To verify the fitting accuracy of the plane equation, discrete points at different positions were acquired using the SECCM, and their actual coordinates were compared with the theoretical values on the fitted plane to evaluate the fitting performance. First, the surface topography was measured using SECCM to obtain the plane equation expressed in Equation (13).

*z* = 0.0118*x* + 0.0605*y* + 49 (13)

**Figure 10:**
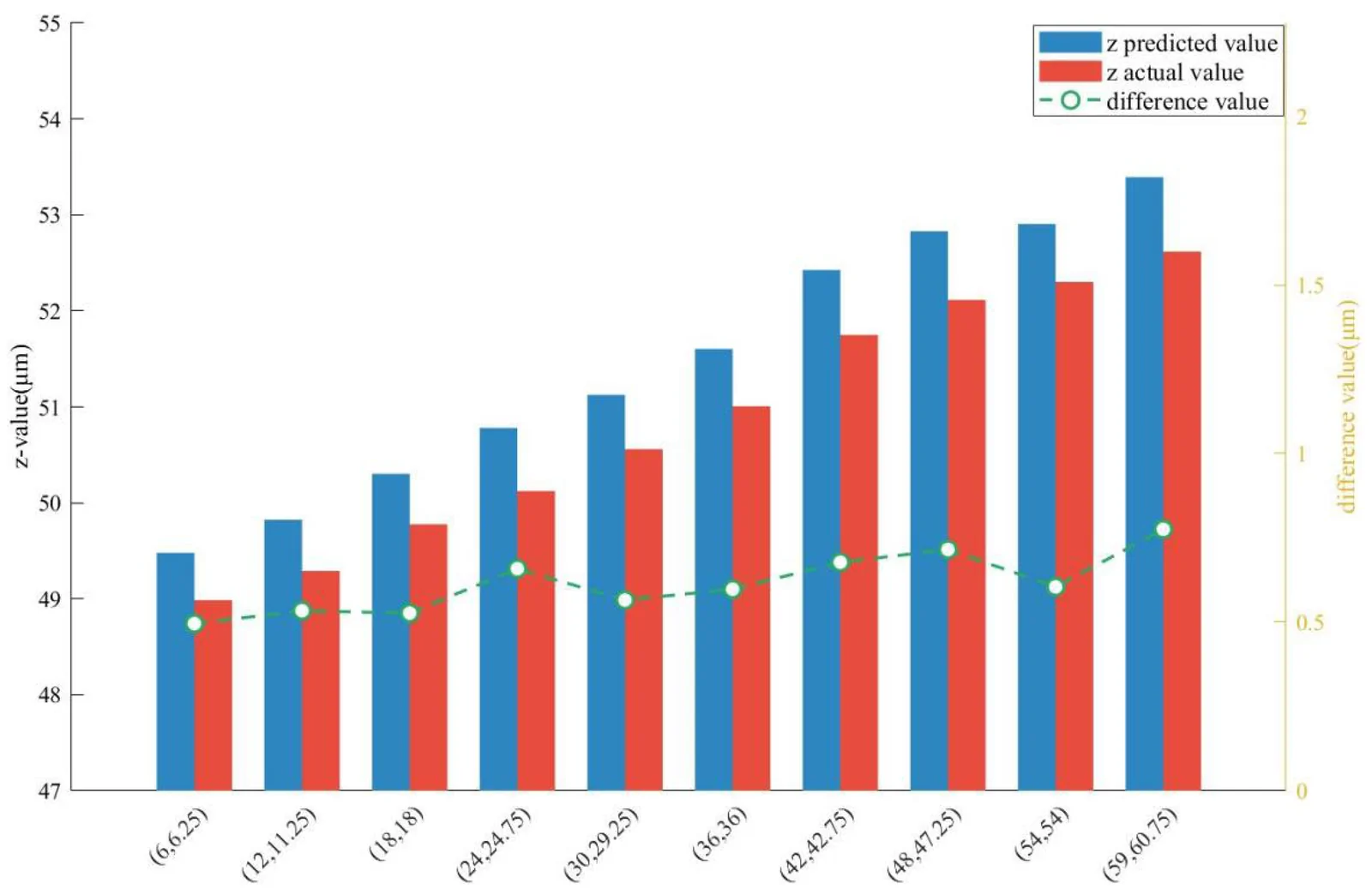
Z-axis coordinate deviation diagram.

Ten discrete points were selected randomly from the deposition planes, as listed in Table 1. The actual Z-axis coordinates were measured using the SECCM, and the fitted coordinate values were calculated using the plane equation. Figure 10 compares the coordinate values and shows a deviation between the fitted and actual values of 0.5–1 μm. Subsequently, the root mean square error (RMSE) was adopted to evaluate the degree of deviation between the fitted plane and the actual point cloud [28].

The calculated *RMSE* value according to Equation (14) was 0.612 μm, indicating that the plane fitting achieved high precision. The minor errors primarily stemmed from the Z-axis reading deviation caused by the current-triggering delay during probe contact. Despite these error sources, the overall fitting results exhibited favorable stability and robustness.

(14) $$\mathrm{RMSE}=\sqrt{\frac{1}{n}{\sum_{i=1}^{n}\left(z_{i}-{\hat{z}}_{i}\right)}^{2}}$$

Here, *Z_i_* represents the actual Z-axis coordinate, and ${\hat{z}}_{i}$ represents the fitted Z-axis coordinate.

### 3.4. Planar Pattern Deposition Experiment

To verify the feasibility of the hybrid deposition path planning algorithm based on Z-axis-weighted adaptive zoning and TSP path optimization, multiple printing experiments on complex planar patterns were conducted. Firstly, the 3D position information within a 70 μm × 70 μm planar area was measured using scanning electrochemical cell microscopy, and the corresponding plane equation was established. The binary image was divided into printing areas, and the printing sequences were optimized using the proposed graphical printing algorithm.

**Figure 11:**
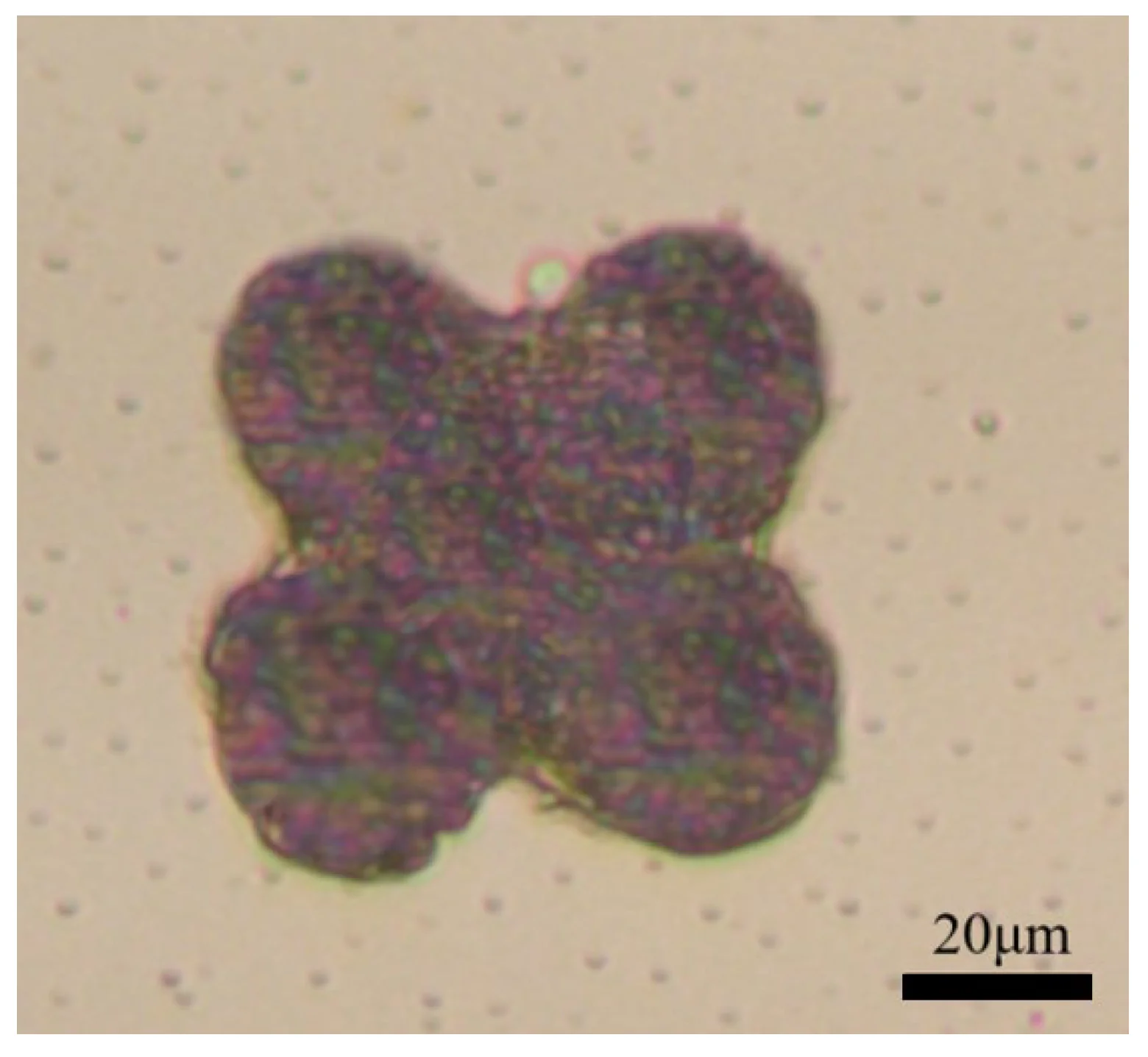
Deposition morphology observed by a metallographic microscope.

The allowable height difference Δ*H_allow_* = 27%·d = 0.27 × 4 μm = 1.08 μm, $K={\left[\frac{\Delta H}{\Delta H_{\mathrm{allow}}}\right]}^{2}$ = 4. Subsequently, 3D deposition paths matching the actual substrate plane were generated to conduct the pattern deposition experiments. The deposited morphologies observed using an XJL-20 inverted metallographic microscope are shown in Figure 11.

The experimental results demonstrate that the proposed ZAWP-TSP algorithm can realize electrochemical deposition fabrication of planar patterns using glass microprobes. A 60 μm × 60 μm nominal pattern is successfully deposited within the 70 μm × 70 μm working area, with a complete geometric structure and visually continuous edges.

**Figure 12:**
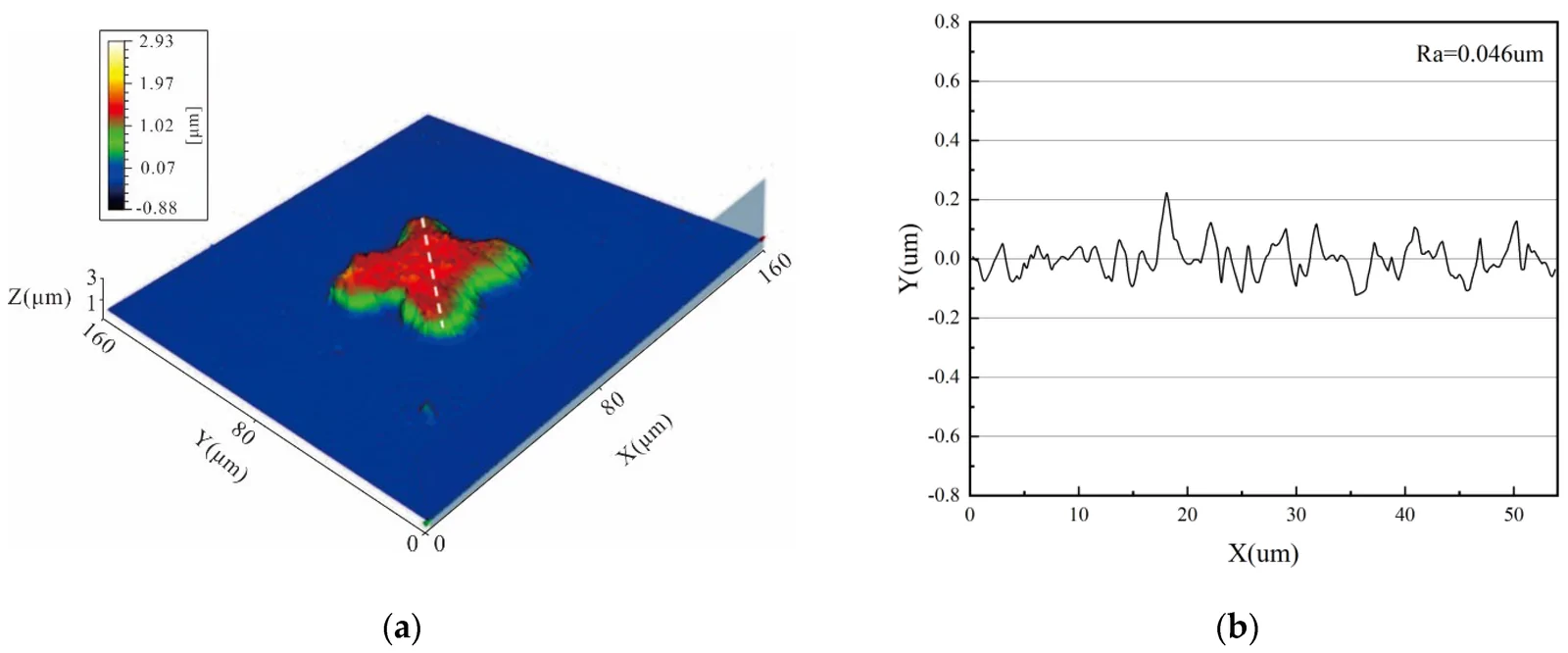
Deposition results observed via a high-resolution confocal microscope. (**a**) Image zoning. (**b**) Cross-sectional curve and average roughness at the white dashed line in (**a**).

Furthermore, high-resolution confocal microscopy was used to perform detailed microscopic imaging analysis of the surface morphology of the deposited samples, thereby comprehensively evaluating the quality and uniformity of the deposited structures. As shown in Figure 12, the confocal images display the surface characteristics of the deposited patterns, providing intuitive evidence for in-depth research on the microscopic morphology. To quantify the flatness and detailed features of the deposited surface, high-precision surface-roughness measurements were performed on the white cross-sectional area shown on the left. The measurement result shows that the surface roughness of this area is only 0.046 μm, indicating extremely tiny microscopic undulations and excellent surface quality of the deposition layer. Note that one representative linear-profile roughness result is presented herein. This profile is selected to verify whether the deposition can meet the key threshold of 0.05 μm, serving as a representative example.

From microscopic observation of Figure 12a, the deposited patterns seem to have a broadly smooth surface, showing small local height variations without evident particle accumulation or significant uneven deposition. These visual observations suggest that the MCED-fabricated pattern exhibits relatively smooth surface morphology, ensuring uniform material distribution during deposition and improving the forming quality and fineness of the deposited structures.

**Figure 13:**
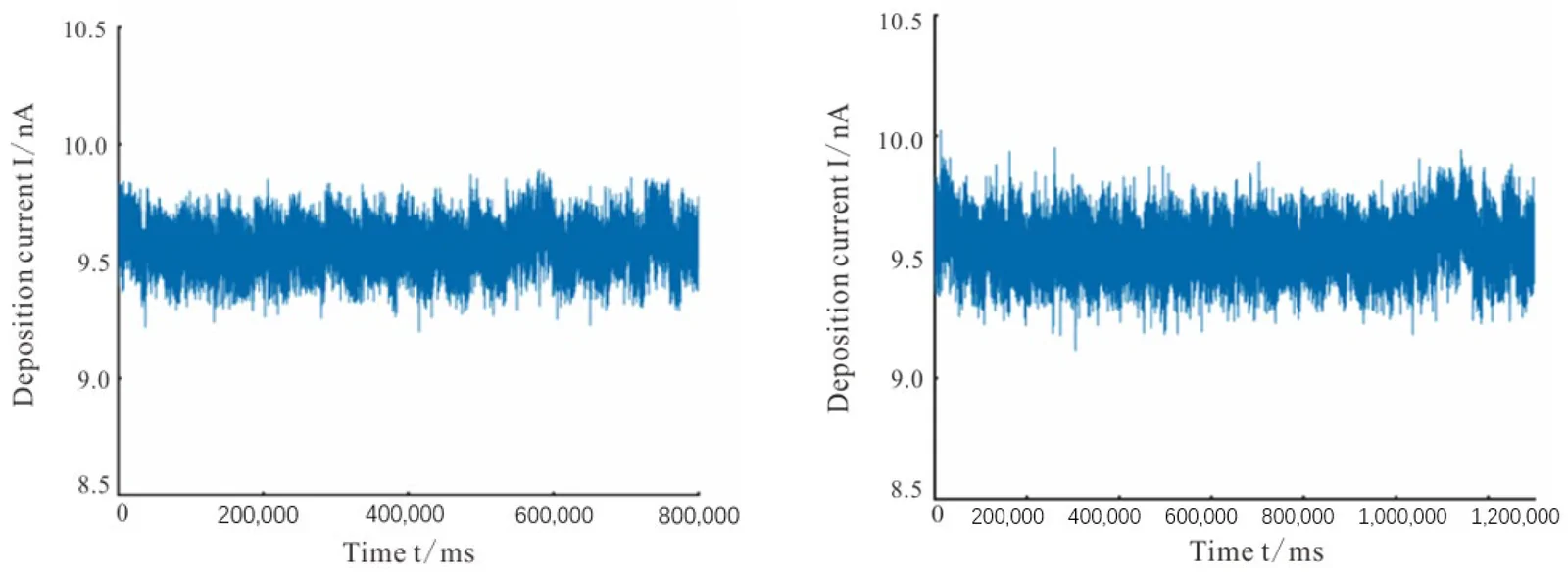
Deposition current comparison diagram. (**a**) Variation in the deposition current in the ZAWP-TSP algorithm. (**b**) Variation in the deposition current in the direct scanning method.

To further verify the superiority of the proposed pattern-deposition algorithm, a comparative analysis was conducted with the existing direct parallel line-scanning printing algorithm. The current variation and time consumption during the deposition of the proposed algorithm are shown in Figure 13a, whereas those of the direct parallel-line scanning printing algorithm are presented in Figure 13b. By comparing the current changes in the deposition processes of the two methods, the advantages of the optimized algorithm in terms of deposition efficiency and current stability can be observed more intuitively.

For fair comparison, the baseline direct parallel-line scanning method uses identical experimental conditions to the proposed ZAWP-TSP. The baseline method adopts a fixed 2 μm line spacing, performs full-field unidirectional raster scanning without region partitioning (region count = 1), and uses fixed return strokes between adjacent lines.

As shown in Figure 13, in this representative experimental case, the algorithm proposed in this paper achieves improved performance in terms of deposition time consumption and current stability. The total deposition time of the nearest neighbor path optimization algorithm is approximately 13.33 min, while that of the direct parallel line deposition method is 22.08 min, shortening the single printing duration.

In terms of current stability, the root mean square error (RMSE) of the deposition current of the optimized algorithm in this test is 0.1052 nA, whereas the RMSE of the direct deposition method is 0.1103 nA. The data indicate that under the tested experimental conditions, this path optimization strategy does not cause a significant reduction in current stability.

In summary, under the tested conditions, the hybrid deposition path planning algorithm based on Z-axis weight adaptive partitioning and Traveling Salesman Problem (TSP) path optimization is expected to alleviate the meniscus instability and printing interruption problems caused by the overall substrate tilt in micro-current electro-deposition (MCED). Deposited patterns with surface roughness less than 0.046 μm were successfully obtained in this representative experiment.

The proposed algorithm demonstrates strong feasibility and practical potential, providing strong technical support for the further development of MCED in micro- and nano-additive manufacturing.

## 4. Conclusions

This paper proposes a hybrid pattern printing algorithm that integrates Z-axis-weighted adaptive pattern partitioning and greedy nearest-neighbor TSP heuristic path optimization. Leveraging the characteristics of MCED printing, the algorithm optimizes adaptive pattern partitioning by weighting Z-axis coordinates and sorting regional paths using a greedy nearest-neighbor TSP heuristic. Experimental validation in this work is performed on nominal-flat gold-coated glass substrates with only global inclination. The plane-fitting procedure compensates for global substrate tilt and cannot handle substrates with local bending, deformation or random surface irregularities. Under the experimental conditions in this study, this method improves the stability and efficiency of planar pattern printing based on MCED, and provides an algorithm reference for subsequent related research on printing three-dimensional microstructures by layer-by-layer stacking using MCED.

The experimental results demonstrate that the ZAWP-TSP printing algorithm enables continuous printing of a 60 μm × 60 μm planar test microstructure using MCED. While ensuring deposition continuity, it also improves the surface quality of the deposited structures. The RMSE of the deposition current is within 0.2 nA, resulting in more uniform deposits with surface roughness below 0.05 μm.

This method features low deposition cost and high forming success rate, and can be used for fabricating various metal microstructures and micro-components within a 70 μm × 70 μm plane where the total height difference of the substrate is less than 2.02 μm.

## Figures and Tables

**Table 1:** Complete data for 10 verification points.

Dot	X Coordinate/μm	Y Coordinate/μm	z Predicted Value	z Actual Value	Difference Value
1	6	6.25	49.45	48.93	0.52
2	12	11.25	49.82	49.27	0.55
3	18	18	50.30	49.76	0.54
4	24	24.75	50.78	50.15	0.63
5	30	29.25	51.10	50.54	0.56
6	36	36	51.60	51.02	0.58
7	42	42.75	52.45	51.81	0.64
8	48	47.25	52.87	52.16	0.71
9	54	54	52.90	52.31	0.59
10	59	60.75	53.37	52.61	0.76

## Data Availability

The original contributions presented in this study are included in the article. Further inquiries can be directed to the corresponding author.

## Nomenclature

**Symbol**	**Definition**	**Unit**
α	Height-weighting factor for clustering feature	Dimensionless
ΔH	Global maximum height difference over working area	μm
ΔHallow	Allowable stable height difference of meniscus	μm
D	Probe tip diameter	μm
d_1_	Meniscus contact diameter on substrate	μm
K	Number of partitioned subregions	Dimensionless
R	Curvature radius of liquid meniscus	μm
r, r_0_	Radial coordinates of the meniscus model	μm
α_0_	Contact angle of the meniscus	rad
zᵢ	Measured Z-axis coordinate of sampling point i	μm
ẑᵢ	Plane-fitted Z-axis coordinate of sampling point i	μm
v_p_	Probe scanning speed	μm/s
